# Composition and Morphology of Nanocrystals in Urines of Lithogenic Patients and Healthy Persons

**DOI:** 10.1155/2009/925297

**Published:** 2009-12-20

**Authors:** Bao-Song Gui, Rong Xie, Xiu-Qiong Yao, Mei-Ru Li, Jian-Ming Ouyang

**Affiliations:** ^1^Department of Nephrology, The Second Hospital of Xi'an Jiaotong University, Xi'an, 710004, China; ^2^Institute of Biomineralization and Lithiasis Research, Jinan University, Guangzhou 510632, China

## Abstract

The composition and morphology of nanocrystals in urines of healthy persons and lithogenic patients were comparatively investigated by means of X-ray diffraction (XRD) and transmission electron microscopy (TEM). It was shown that the main composition of urinary nanocrystals in healthy persons were calcium oxalate dihydrate (COD), uric acid, and ammonium magnesium phosphate (struvite). However, the main compositions of urinary nanocrystals in lithogenic patients were struvite, *β*-tricalcium phosphate, uric acid, COD, and calcium oxalate monohydrate (COM). According to the XRD data, the size of nanocrystals was calculated to be 23∼72 nm in healthy urine and 12∼118 nm in lithogenic urine by Scherer formula. TEM results showed that the nanocrystals in healthy urine were dispersive and uniform with a mean size of about 38 nm. In contrast, the nanocrystals in lithogenic urine were much aggregated with a mean size of about 55 nm. The results in this work indicated that the urinary stone formation may be prevented by diminishing the aggregation and the size differentiation of urinary nanocrystals by physical or chemical methods.

## 1. Introduction

Urolithiasis, which occurs in 1 ~ 20% in Asia, 5 ~ 9% in Europe, and approximately 13% in North America, is a worldwide common and frequently occurring disease [1, 2]. Especially, it has high incidence in adult men among 20 ~ 40 years old who have large amount of exercise. The main components of urinary stones include calcium oxalate, calcium phosphate, uric acid, magnesium ammonium phosphate (struvite), and cystines. Approximately 80% of stones are calcic in which calcium oxalate is the main component [2–5].

The first step of urinary stone formation is the nucleation of particles in supersaturated urine. Then the formed nuclei (generally less than 10 nm) grow or/and aggregate to a pathological size (several tens micron). After these crystallites are retained on the urinary tract (free-particle theory) or fixed by the urinary tract organization (fixed-particle theory), urinary stones ultimately formed [6].

According to the chemical properties, urinary stone can be classified into acidic stones (such as uric acid, cystine), alkaline stones (such as struvite), and neutral stones (such as calcium oxalate, calcium phosphate). However, clinical practices neither can be predicted before stone formation nor can be individually treated by taking different medicine and different treatment methods for different types of urinary stones. Thus it results in a low treatment ratio. Thereby, if the occurrence of urinary stones can be predicted or the type of stones can be estimated before treatment by the determination of urinary composition and urinary nanocrystals, it will provide evidence for a suitable remedy and personalized treatment in clinic.

XRD has advantages of reliability in qualitative analysis and accuracy in quantitative analysis. It operates simply and has a high sensitivity. Based on XRD diffraction data, the multicomponents in a sample can be measured simultaneously, and the approximate size of the nanoparticles can also be calculated by means of Scherer formula. For example, Chen et al. [7] had calculated the size of nanosilver (Ag) to be 7–22 nm, and Satyanarayana et al. [8] had calculated the size of nanocuprous oxide (Cu_2_O) to be 11 nm by Scherer formula. Transmission electron microscopy (TEM), with larger amplification and high resolution, is commonly used in the observation of nanomaterials. The examination of urinary nanocrystals by means of TEM can help us to understand the relationship between these nanocrystals and formation of various types of urinary stones.

 Based on the discussed above, the composition, size, morphology, and aggregation of the nanocrystals of less than 100 nm in healthy urines and in lithogenic urines are comparatively investigated by XRD and TEM. We hope it would provide enlightenment on diagnosing and treating urinary stone.

## 2. Experimental Details

### 2.1. Materials

Anhydrous alcohol and sodium azide were reagent-grade chemicals. All the glasswares were cleaned with the ultrapure water from the Millipore-Q system; the resistance was 18.2 MΩ cm. The fresh morning urine samples were collected from both patients who had urinary stones and healthy persons with no prior history of urinary stones.

### 2.2. Apparatus

TEM was carried out by PHILIPS TECNAI-10 transmission electron microscope at an operating voltage of 100 kV. X-ray diffraction (XRD) results were recorded on a D/max 2400 

X-ray diffractometer (Rigaku, Japan) used graphite monochromator, Ni-filtered Cu K*α* radiation (*λ* = 0.15406 nm), and a scanning rate of 0.02°*s* ^−1^ at 40 kV, 30 mA. The divergence and scattering slit was 1*º* for the range 5° <2*θ* <60°. Urinary stones and urinary crystals were identified according [9]. Magnesium ammonium phosphate monohydrate (MgNH_4_PO_4_ · H_2_O) and hexahydrate (MgNH_4_PO_4_ · 6H_2_O, struvite) were compared with ASTM card number 20-0663 and 77-2303, respectively. Octacalcium phosphate (Ca_8_H_2_(PO_4_)_6_ · 5H_2_O), calcium phosphate (Ca_3_(PO_4_)_2_, CaP), and hydroxylapatite (HAP) were compared with the number 44-0778, 32-176, and 09-0432, respectively. Uric acid (UA), calcium oxalate monohydrate (COM), and dihydrate (COD) were compared with the number 09-0432, 20-231, and 17-541, respectively.

### 2.3. Clinical Data and Test Method

#### 2.3.1. Clinical Data

**Figure 1:**
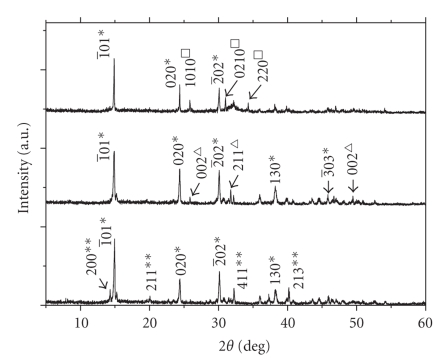
XRD patterns of three representative urinary stones (∗: COM, ∗∗: COD, △: HAP, □: Ca_3_(PO_4_)_2_).

The participants in the study included sixteen lithogenic patients (9 men and 7 women) with a mean age of 44.7 years (23 ~ 70 years old) and seventeen random selected healthy human (8 men and 9 women) with no prior history of urinary stone with a mean age of 38.6 years (22 ~ 53 years old). The urinary stones were collected after surgery, cleared by double distilled water, and put in a no dust incubator about 50°C to make dry, then grinded into powder by agate mortar for XRD and FT-IR characterization. The results showed that the main compositions of these stones were CaOxa or CaOxa–CaP. The XRD patterns of three representative urinary stones were shown in Figure 1. The fresh morning urine from the patients with CaOxa stone or CaOxa–CaP stone was collected from 06:00 to 08:00 AM. without being submitted to special diets. Peoples who provided urine samples did not eat any food from 9:00 PM. last night to acquisition time of their urine samples. Table 1 showed the urinary stone risk profile in urine of lithogenic patients and normal subjects.

#### 2.3.2. Test Method

After the fresh morning urine was collected, 2% (weight/volume ratio) NaN_3_ solution (urine : NaN_3_ = 100 : 1 in volume) was immediately added into the urine as antiseptic. Then the urine was added anhydrous alcohol (urine: anhydrous alcohol = 3 : 2 in volume) and was stood for half hour to make the urinary protein denaturation. The cell debris and the denatured protein precipitations were filtered out using a microporous membrane with the pore size of 1.2 *μ*m. It was shown that the filtration with a 1.2 *μ*m microporous membrane had significant influence on the particles larger than 400 nm and exerted little influence on the small particles with the size less than 100 nm in the two kinds of urines.

 The filtrate was added on a copper mesh for TEM detection and on clean glass tablet for XRD characterization simultaneously. For the former, the copper mesh was put in a desiccators, and for the latter, the glass tablet was put in a no dust incubatorat at 50°C for 4 hours to make urine evaporation.

## 3. Results and Discussion

### 3.1. Differentiation of Components of Urinary Nanocrystals in Lithogenic Urine and in Healthy Urine

**Figure 2:**
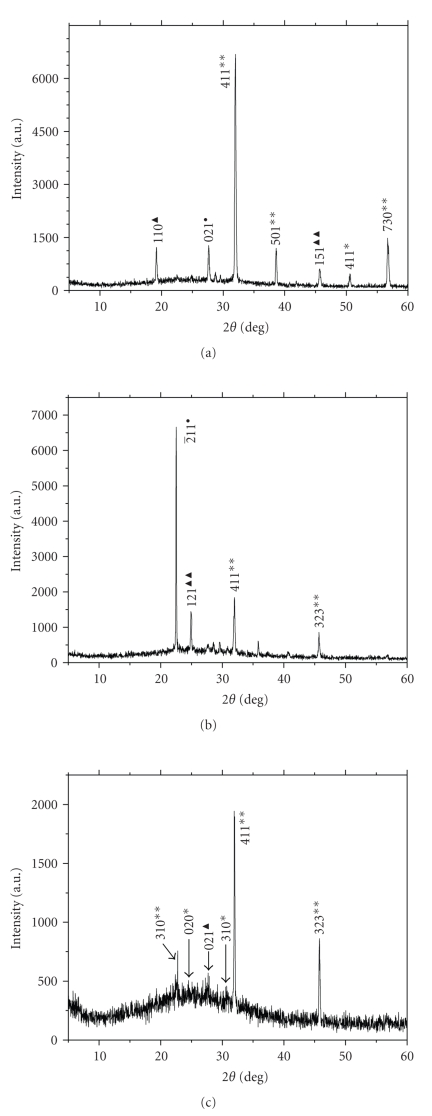
XRD patterns of typical urinary crystals of three health persons (H1, H2, H3). (∗: COM, ∗∗: COD, ●: uric acid, ▴: MgNH_4_PO_4_ · H_2_O, ▴ ▴: MgNH_4_PO_4_ · 6H_2_O).

**Figure 3:**
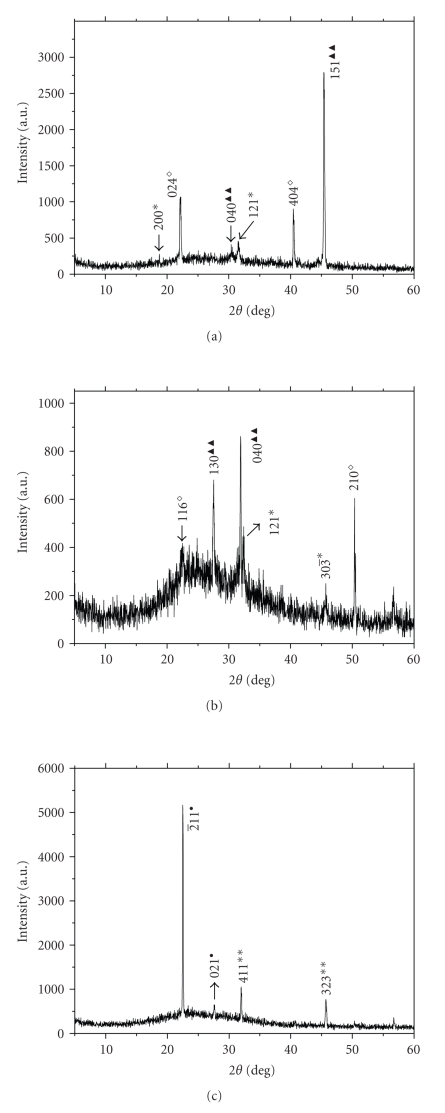
XRD patterns of typical urinary crystals of three lithogenic patients (L1, L2, L3). (∗: COM, ∗∗: COD, ●: uric acid, ▴ ▴: MgNH_4_PO_4_ · 6H_2_O, ◇: *β*-Ca_3_(PO_4_)_2_).

XRD was used to investigate the composition of urinary nanocrystals of 16 lithogenic patients and 17 healthy persons. Figures 2 and 3 showed the representative XRD patterns of the urinary nanocrystals in three healthy persons and in three lithogenic patients, respectively. Compared with the standard diagrams (see Table 2) [9], the main peaks in Figure 2(a) were assigned to the (110) crystal plane of MgNH_4_PO_4_ · H_2_O, the (021) plane of uric acid (411), (501), and (730) planes of calcium oxalate dihydrate (COD). The main peaks in Figure 2(b) were assigned to the ($\bar{2}11$) plane of uric acid, the (121) plane of MgNH_4_PO_4_ · 6H_2_O, and the (411) and (323) planes of COD crystals. The main peaks in Figure 2(c) were assigned to the (310), (411), and (323) planes of COD crystal. That is, all the three urinary nanocrystals in healthy persons contained COD crystals.

In the three XRD patterns of urinary nanocrystals of lithogenic patients (see Figure 3), the main peaks in Figure 3(a) were, respectively, assigned to the (024) and (404) crystal planes of *β*-Ca_3_(PO_4_)_2_ and the (040) and (151) planes of MgNH_4_PO_4_ · 6H_2_O. The main peaks in Figure 3(b) were assigned to the (130) and (040) planes of MgNH_4_PO_4_ · 6H_2_O, the (121) plane of COM, and the (210) plane of *β*-Ca_3_(PO_4_)_2_, respectively. In Figure 3(c) the main peaks were, respectively, assigned to the $\left(\bar{2}11\right)$ and (021) planes of uric acid, and the (411) and (323) planes of COD crystals_._

All the XRD results showed that the urinary nanocrystals were mainly COD, uric acid and magnesium ammonium phosphate in healthy urine (see Figure 2) and were mainly magnesium ammonium phosphate, *β*-tricalcium phosphate, COD, and COM in lithogenic urine (see Figure 3).

It could be seen that the peak intensities of struvite crystals in urine of lithogenic patients (see Figure 3(a)) were stronger than those in urine of healthy persons (see Figures 2(a)-2(b)). It was attributed to that the concentration of citrate in health urine (2.32 ± 0.85 mmol/L) was higher than that in lithogenic urine (1.71 ± 0.94 mmol/L) as shown in Table 1 and as reported by Biyani et al. [10]. Citrate could form complexes with phosphoric acid or phosphate in urine, therefore inhibited struvite precipitation [11].

It could also be seen that the peak intensities of (411) and (323) crystal planes of COD crystals in urine of healthy persons (see Figures 2(a)-2(c)) were stronger than those in urine of lithogenic patient (see Figure 3(c)). Since the diffraction peak intensity was positively related with the number of crystals, it indicated that the amount of COD crystals in healthy urine was more than that in lithogenic urine.

The differentiation of COD crystals in the two kinds of urine was attributed to the difference of urinary inhibitors. These urinary inhibitors could inhibit the formation of COM crystals and induce the growth of COD crystals [12–14]. One kind of these inhibitors was small molecule substance such as citrate, glutamic acid, pyrophosphate, magnesium, and the other kind was the urinary macromolecules such as glycosaminoglycans (GAGs), uromucoid, prothrombin fragment F1 (UPTF1), Tamm-Horsfall protein, nephrocalcin (NC), osteopontin (OPN), and polyribonucleotide [10, 15, 16]. These substances, especially the urinary macromolecules, had much negative charges and were inclined to be the nucleated nidus for urinary stone such as CaOxa stone [17, 18]. For the bipyramidal COD crystals, there were only high-density charged locate at the two vertexes in which the Oxa^2-^ ions were outstanding and negatively charged. However, the surfaces of COM crystals were positively charged [19]. So the negatively charged urinary macromolecules preferentially combined with the face of COM crystals, which is positively charged [20–22] and blocked the growth sites on the surface of COM. It increased the negative value of Zeta potential on the surface of COM crystals and led to a stronger repellent action among the nanocrystals in urine. Thus, the nucleation and growth of COM crystals in healthy urine were inhibited. However, in urine of lithogenic patients, either the concentration of macromolecules was less or their activity was lower. For example, the concentration of GAGs in urine of healthy human was about 8.80 ± 1.92 mg/L; however, it decreased to about 6.08 ± 1.39 mg/L in lithogenic urine. Therefore, the interaction between the urinary macromolecules and nanocrystals was weaker in urine of lithogenic patients. Thus less COD crystals were induced. That is, calcium oxalate mainly precipitated as COM crystals in the urine of lithogenic patients. It made the intensity of diffraction peaks of COD crystals in healthy urine generally be stronger than that in lithogenic urine.

### 3.2. Differentiation of Morphology and Aggregation of Urinary Nanocrystals in Lithogenic Urine and in Healthy Urine

**Figure 4:**
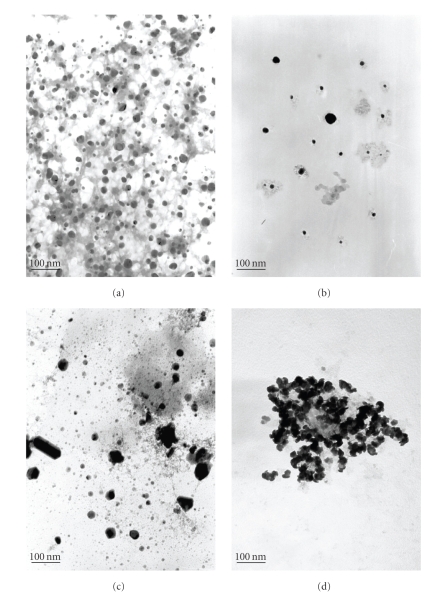
TEM images of typical urinary nanocrystals of two health persons (a), (b) and two lithogenic patients (c), (d). The bar is 100 nm.

Figure 4 showed the TEM images of representative urinary nanocrystals of healthy persons and lithogenic patients. The urinary nanocrystals of healthy persons were well dispersed and uniform with a size distribution between 15 nm and 60 nm (see Figures 4(a), 4(b)). In contrast, the nanocrystals of lithogenic patients were not uniform with a size distribution ranging from 10 nm to more than 100 nm (see Figures 4(c), 4(d)). In addition, more aggregated nanocrystals were found in lithogenic urine.

Based on the XRD diffraction data, not only could the components of samples be measured, but also the approximate size of the nanoparticles could be calculated using Scherer formula [7, 8, 23] as shown in (1):

(1) $$L=\frac{K\lambda }{\cos\;\theta \cdot \Delta \left(2\theta \right)},$$

where *L *was the size of the nanoparticles, K is constant and is dependent on crystallite shape (0.89), *λ* was the X-ray wavelength (*λ* = 1.5418 Å in this experiment), *∆*(2*θ*) was the radian value of fwhm (full width at half-max), and *θ* was the Bragg angle. For example, in Figure 2(a), the strongest characteristic diffraction peak of COD crystals was the (411) face with a *d* value of 0.279 nm, the corresponding 2*θ* = 32.00° and *θ* = 16.00°. The maximal intensity of this peak was 6689.2, so the height at half-max was 3344.6, and the corresponding 2*θ* values at the two points of half-max were 32.09° and 31.86°, respectively. That was ∆(2*θ*)＝(32.09° −31.86°) · *π*/180°. By substituting these values into (1), we could figure out that the size of COD nanocrystals *L*(COD) was 41 nm. By means of the same method, we could also figure out the size of the other nanocrystals in healthy urine. For example, for the sample H1 in Figure 2(a), *L *(MgNH_4_PO_4_ · H_2_O) = 32 nm (110 face), *L*(uric acid)*  * = 23 nm (021 face), and *L*(COD) = 41 ~ 47 nm ((411), (501), and (730) faces). For the sample H2 in Figure 2(b), *L*(COD) = 37 ~ 49 nm ((411) and (323) faces), *L*(uric acid)*  * = 72 nm ($\bar{2}11$ face), *L*(struvite)*  * = 40 nm (121 face). For the sample H3 (Figure 2(c)), *L*(COD) = 45 ~ 51 nm ((310), (411), and (323) faces) (see Table 3).

For the urinary nanocrystals in lithogenic urine sample L1 (Figure 3(a)), it could be calculated that the size of nano-*β*-Ca_3_(PO_4_)_2_ (*L *(*β*-Ca_3_(PO_4_)_2_)) was 29 ~ 45 nm ((024) and (404) faces); *L*(MgNH_4_PO_4_ · H_2_O) = 51 nm ((040) and (151) faces). For sample L2 (Figure 3(b)), *L *(*β*-Ca_3_(PO_4_)_2_) = 118 nm ((210) face), and *L*(struvite) = 12 ~ 39 nm ((130) and (040) faces). For sample L3 (Figure 3(c)), *L*(uric acid) = 27 ~ 65 nm (($\bar{2}11$) and (021) faces), and *L*(COD) = 36 ~ 45 nm ((411) and (323) faces).

That is, the size of nanocrystals in the healthy persons urine ranged from 23 nm to 72 nm, and those in lithogenic patients urine ranged from 12 nm to 118 nm. The results obtained from Scherer formula were nearly consistent with those observed from the TEM method.

Since there was a different ratio of volume to surface area for the particles with different size, it led to the difference in their solubility. According to the mechanism of Ostwald ripening [24], when solid particles were dispersed in their own saturated solution, there was a tendency that the small-size particles be dissolved and the solute be deposited subsequently on the large-size particles. Therefore, the uneven distribution of nanoparticles in lithogenic urine made the small-size crystals disappeared and the large-size crystals grew; thus these urine samples were unstable. However, the nanoparticles in healthy urine were uniform. It led to a weak interaction and a small difference of the surface free energy between particles; thus the driving force for these nanoparticles to aggregate diminished and finally these urine samples were stable.

**Figure 5:**
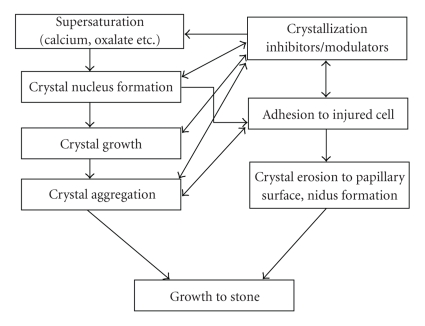
Schematic diagram of urinary stone formation.

Although the formation of urinary stones correlated with many factors, it necessarily went through the following processes: the formation of crystal nucleus in urine, the growth and/or aggregation of the crystallites, and the adhesion of crystallites on renal epithelial cell, as shown in Figure 5. According to the calculation results of crystal growth rate and flow rate of urine through tubule lumen, a single crystallite could not block the tubule lumen badly when it passed the nephron. Only after the urine crystallites had combined with other substances such as the cell debris in urine, the aggregated crystallites were formed, then the larger size of crystals could block the nephron, and finally stone formed [25]. 

Since the growth, aggregation and the final formation of urinary stones were apparently affected by the components, size, and uniformity of urinary nanocrystals, therefore, urinary stone formation may be prevented by diminishing the size differentiation and the aggregation of nanocrystals in urine through physical or chemical methods. The investigatation about the existing state of urinary nanocrystals and the detection of the components and properties of nanocrystals in urine would be beneficial to find out the connection between stone formation and urinary nanocrystals and then to provide evidences for a suitable remedy and personalized treatment to urolithiasis in clinic.

## 4. Conclusion

The composition and morphology of nanocrystals in urines of healthy persons and lithogenic patients were comparatively investigated by means of XRD and TEM. XRD patterns showed that the urinary nanocrystals mainly were COD, uric acid, and magnesium ammonium phosphate in healthy persons; but mainly were magnesium ammonium phosphate, *β*-tricalcium phosphate, COD, and COM in lithogenic patients. Especially the amount of COD crystals in healthy urine was much more than that in lithogenic urine. Based on the XRD diffraction data, the size of urinary nanocrystals was figured out to be 23 ~ 72 nm in healthy urine and about 12 ~ 118 nm in lithogenic patients, respectively, by means of Scherer formula. TEM results showed that the nanocrystals in healthy urine were dispersive and uniform with an average size of about 38 nm. In contrast, the nanocrystals in lithogenic urine were much aggregated with an average size of about 55 nm. It was consistent with the results calculated from XRD data. The results showed that the urinary stone formation may be prevented by diminishing the size differentiation and the aggregation of nanocrystals in urine through physical or chemical methods.

## Figures and Tables

**Table 1:** Urinary stone risk profile in lithogenic patients and in normal subjects.

Urine parameter	Lithogenic patients (*n* = 16)	Normal subjects (*n* = 17)
Volume, L	1721 ± 429	1202 ± 354
pH	6.32 ± 0.35	6.02 ± 0.32
Calcium, mmol/L	5.51 ± 1.32	5.71 ± 2.11
Oxalate, mmol/L	0.53 ± 0.09	0.29 ± 0.05
Citrate, mmol/L	1.71 ± 0.94	2.32 ± 0.85
Phosphorus, mmol/L	15.3 ± 5.8	16.1 ± 4.7

**Table 2:** Main diffraction peaks (*d* value) and corresponding crystal faces of urinary nanocrystals.

Nanocrystal in urine	*d* value (crystal plane)	ASTM card [9]
NH_4_MgPO_4_ · H_2_O	8.79(010), 2.80(121), 4.73(110), 4.20(011), 3.24(012)	36-1491
NH_4_MgPO_4_ · 6H_2_O	5.60(020), 3.66(121), 3.29(130), 2.80(040), 2.01(151)	15-762
C_5_H_4_N_4_O_3_	4.91(210), 3.86$\left(\overset{̅}{2}11\right)$, 3.19(021), 3.10$\left(\overset{̅}{1}21\right)$	31-1982
*β*-Ca_3_(PO_4_)_2_	4.06(024), 4.00(116), 2.88(217), 2.20(404), 1.81(210)	9-169
CaC_2_O_4_ · H_2_O(COM)	5.93$\left(\overset{̅}{1}01\right)$, 3.65(020), 2.97$\left(\overset{̅}{2}02\right)$, 2.84(121), 1.98$\left(\overset{̅}{3}03\right)$	20-231
CaC_2_O_4_ · 2H_2_O(COD)	6.18(200), 4.42(211), 3.91(310), 2.78(411), 2.34(501),	17-541
	2.24(213), 2.00(323), 1.62(730)	

**Table 3:** Sizes of urinary nanocrystals in three healthy persons (H) and in three lithogenic patients (L) calculated using scherer formula (nm).

Urine nanocrystals	H1	H2	H3	L1	L2	L3
COD	41 ~ 47	37 ~ 49	45 ~ 51	—	—	36 ~ 45
struvite	32	40	—	—	—	—
uric acid	23	72	—	—	—	27 ~ 65
*β*-Ca_3_(PO_4_)_2_	—	—	—	29 ~ 45	118	—
struvite	—	—	—	51	12 ~ 39	—
